# Expression of Selected Pharmacologically Relevant Transporters in Murine Non-Parenchymal Liver Cells Compared to Hepatocytes

**DOI:** 10.3390/ijms262211116

**Published:** 2025-11-17

**Authors:** Vincent Rönnpagel, Anett Ullrich, Christy Joseph, Mladen V. Tzvetkov, Dieter Runge, Markus Grube

**Affiliations:** 1 Institute for Pharmacology, Center of Drug Absorption and Transport (C_DAT), University Medicine Greifswald, Felix-Hausdorff-Str. 3, 17487 Greifswald, Germany; vincent.roennpagel@med.uni-greifswald.de (V.R.);; 2 PRIMACYT Cell Culture Technology GmbH, 19061 Schwerin, Germany

**Keywords:** non-parenchymal liver cells, drug transporter, membrane transporters, mouse, hepatocytes, endothelial cells, Kupffer cells

## Abstract

Primary hepatocytes are widely used in preclinical drug development, with their transporter expression being well-characterized. However, less is known about non-parenchymal liver cells (NPCs), which constitute 40% of the liver’s cell population and include sinusoidal endothelial cells and Kupffer cells. This study aimed to characterize transporter expression in murine NPCs compared to hepatocytes. Cell fractions were isolated using collagenase perfusion, density gradient centrifugation, and magnetic-activated cell sorting (MACS) with F4/80 and CD146 antibodies. Transporter expression and separation quality were analyzed via RT-qPCR. Results showed NPC-specific genes were significantly lower in hepatocytes and vice versa. Importantly, NPCs exhibited higher expression of several transporters: Abcc1/Mrp1 (87-fold), Abcc4/Mrp4 (4-fold), Abcc5/Mrp5 (40-fold), as well as Slc15a2/PepT2 (16-fold), Slc28a2/Cnt2 (20-fold), Slco3a1/Oatp3a1 (15-fold), and Slco4a1/Oatp4a1 (13-fold), compared to hepatocytes. Hepatocytes showed dominant expression of Abcc2/Mrp2, Abcg2/Bcrp, Slc22a1/Oct1, and others. Minimal differences in transporter expression were found between Kupffer and endothelial cells. In conclusion, the efflux transporters Abcc1/Mrp1 and Abcc5/Mrp5 are predominantly expressed in NPCs. This suggests that NPCs are potentially relevant for the transport of certain drugs and should be included in in vitro preclinical testing.

## 1. Introduction

The liver consists of approximately 60 to 80% parenchymal cells (PCs) and 20 to 40% non-parenchymal cells (NPCs) [1,2,3]. Among the NPCs, there are 50% liver sinusoidal endothelial cells (ECs), 20% Kupffer cells (KCs), 5% cholangiocytes, and 1% hepatic stellate cells (HSCs) [4]. The remaining cells are tissue-migrated immune cells, including T-, B-, and NK cells [5].

The parenchymal cells, known as hepatocytes, are the primary functional units of the liver and play a crucial role in biotransformation. Hepatocytes are capable of both uptake and efflux of endogenous and exogenous compounds [6]. Primary hepatocytes represent the gold standard for in vitro drug interaction studies, particularly when examining transporter or cytochrome P450 enzyme-mediated drug interactions [7,8]. Given the pharmacokinetically relevant transporters and metabolic enzymes, hepatocytes are very well-characterized [9,10,11,12]. The same applies to the expression of nuclear receptors like the pregnane X receptor (NR1I2/PXR) or the constitutive androstane receptor (NR1I3/CAR) as important regulators for the expression of drug transporters and metabolizing enzymes [13,14,15,16].

In contrast to hepatocytes, NPCs have more immunological and recovery functions [17], and both Kupffer and endothelial cells are the connection between the blood and the hepatocytes [18]. From the pharmacology and toxicology point of view, NPCs are the first liver cells exposed to drugs and toxins and therefore are exposed to high concentrations of these substances [19,20]. NPCs play an important role in drug-induced liver injury (DILI) [17,21]. While in this context the importance of drug transporters in hepatocytes has already been demonstrated, for example, the uptake of hepatotoxic compounds like the pyrrolizidine alkaloid monocrotaline is OCT1-dependent [22], and OATP1B1/1B3 mediates the hepatic uptake of the mushroom toxins phalloidin and amanitin [23,24,25], relatively little is known about such processes in NPCs. In this context, the first step is to characterize the expression of the corresponding genes in these cells in comparison to their expression in hepatocytes.

The aim of this study was to investigate the expression of pharmacologically relevant drug transporters of the ABC- and SLC-family in NPCs, in particular Kupffer and endothelial cells, and compare it to the expression in hepatocytes. This is a prerequisite for elucidating the significance of membrane transporters in NPCs.

## 2. Results

### Characterization of the Quality of the Isolated Cell Fractions

**Figure 1:**
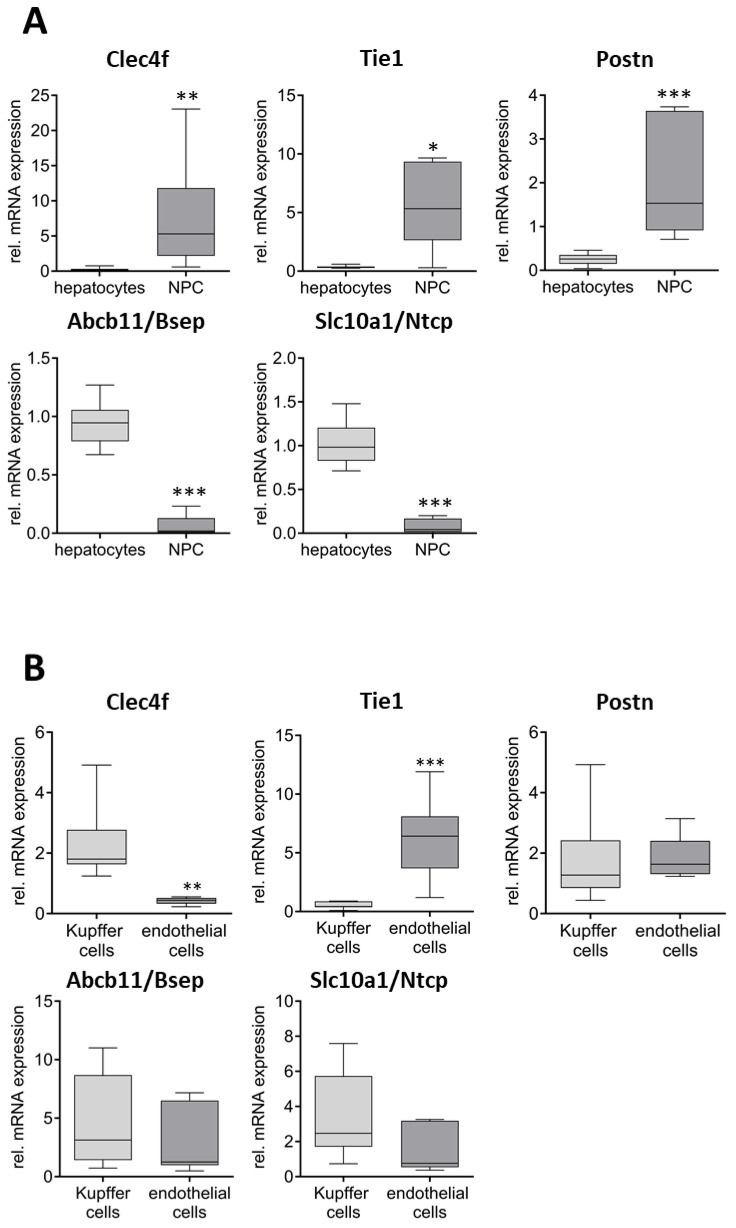
Characterization of cell fraction purity using RT-qPCR. (**A**) Comparison of specific marker expression in hepatocytes (*n* = 7–8) and non-parenchymal cells (NPCs, *n* = 6) relative to expression in whole liver samples after digestion (*n* = 8–9). (**B**) Comparison of marker expression in isolated Kupffer (*n* = 7–10) and endothelial cells (*n* = 6–7) relative to expression in NPCs prior to MACS separation. Specific markers for hepatocytes (Abcb11/Bsep and Slc10a1/Ntcp), Kupffer cells (Clec4f), endothelial cells (Tie1), and stellate cells (Postn) were used for analysis. Statistical analyses were performed using the Mann–Whitney test (* *p* < 0.05, ** *p* < 0.01, *** *p* < 0.001).

First, isolated cell fractions were analyzed for cell-type-specific marker expression to determine the efficiency of separation. For hepatocytes, the mRNA expression of Abcb11/Bsep and Slc10a1/Ntcp was determined, while Clec4f was used as a Kupffer cell and Tie1 as an endothelial cell marker. The presence of stellate cells in the respective fractions was determined by measuring the expression of Postn. As shown in Figure 1A, Abcb11/Bsep and Slc10a1/Ntcp expression was 15- and 13-fold lower, respectively, in the NPC fraction compared to the hepatocytes. In contrast, the expression of NPC-specific markers Clec4f (Kupffer cells), Tie1 (endothelial cells), and Postn (stellate cells) were 27-, 14-, and 8-fold higher in the NPC fraction compared to the hepatocytes. The marker gene expression in the Kupffer and endothelial cell fractions separated by the MACS system was compared to the expression in the NPC fraction. As shown in Figure 1B, Clec4f mRNA was 5-fold higher expressed in Kupffer cells compared to endothelial cells, while Tie1 mRNA was 13-fold higher in the endothelial cell fraction compared to Kupffer cell one, indicating a strong enrichment in both cases. For the control, the expression of the stellate cell marker Postn as well as the hepatocyte markers Abcb11/Bsep and Slc10a1/Ntcp was measured, indicating no significant differences concerning a contamination with these cell types between Kupffer cells and endothelial cells.

**Figure 2:**
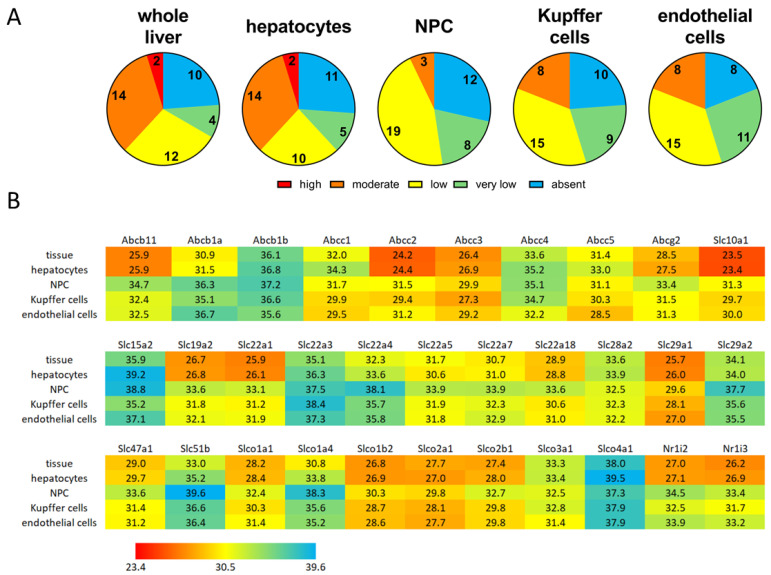
Transporter and nuclear receptor expression in the different cell fractions. (**A**) Self-defined gene expression levels in the whole liver, hepatocytes, NPCs, Kupffer cells, and endothelial cells. Genes with Ct-values between 20 and 25 were considered to be highly expressed; Ct-values between 25 and 30 indicated moderate expression; Ct-values between 30 and 35 indicated low expression; and Ct-values between 35 and 40 indicated very low expression. Genes with no Ct-values were defined as absent. (**B**) Heatmap of the Ct-values (geometric mean) of all measured genes in the different fractions (tissue = whole liver after digestion).

Figure 2A shows the gene expression levels in the different cell fractions. The expression of different target genes (transporter and nuclear receptors) ranged between Ct-values of 23.4 and 40 (absent). Based on these Ct-values, the expression levels were categorized as follows: Ct-values from 20 to 24.9 (high expression); 25 to 29.9 (moderate expression); 30 to 34.9 (low); and between 35 to 40 (very low). All undetectable genes were defined as absent. The absolute transporter expression levels in the different cell fractions based on the geometric mean of the Ct-values are depicted in Figure 2B. The figure shows that in NPCs the strongest expressed transporters were the Slco family members Slco1b2/Oatp1b2, Slco2a1/Oatp2a1, and Slco2b1/Oatp2b1, as well as the Abcc1/Mrp1, Abcc2/Mrp2, and Abcc3/Mrp3. But in general, hepatocytes have stronger transporter expression than the NPCs. This is especially true for bile acid transporters like Ntcp/Slc10a1 and Bsep/Abcb11, but also for a number of potential drug transporters like Abcc2/Mrp2, Abcg2/Bcrp, Slc22a1/Oct1, and Slc19a2/ThTr1. Very low expression levels of Slc15a2/PepT2, Slc22a3/Oct3, Slc29a2/Ent2, Slc51b/Ostβ, and Slco4a1/Oatp1 were consistently observed across all cell fractions.

**Figure 3:**
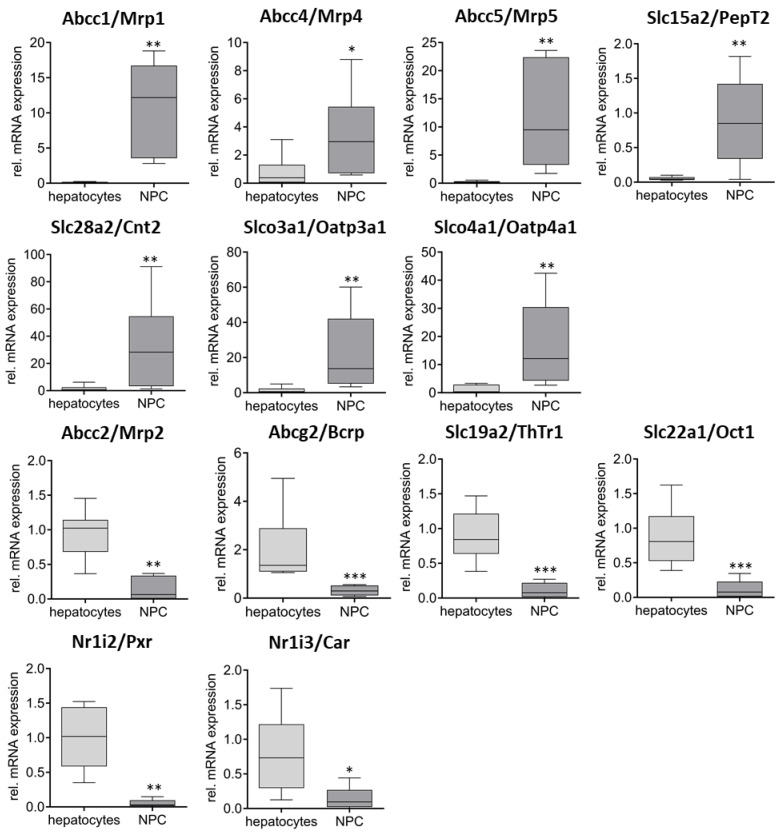
Significantly differentially expressed transporters in hepatocytes and NPCs. The data were presented in relation to the expression in whole liver samples (*n* = 8–9) after digestion. Statistical analyses were performed using the Mann–Whitney test between hepatocytes (*n* = 6–8) and NPCs (*n* = 5–6) (* *p* < 0.05, ** *p* < 0.01, *** *p* < 0.001).

**Figure 4:**
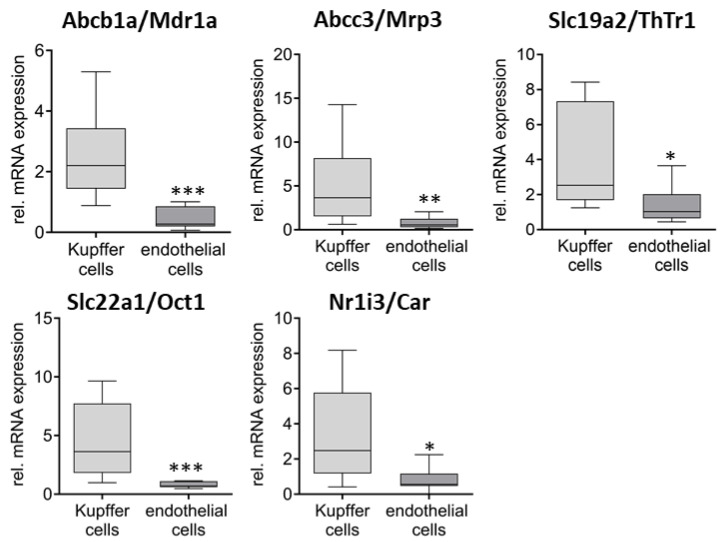
Significantly differentially expressed transporters in Kupffer and endothelial cells. The data were presented in relation to the expression in NPCs (*n* = 5–6) before separation. Statistical analyses were performed using the Mann–Whitney test between Kupffer (*n* = 9–10) and endothelial cells (*n* = 6–7) (* *p* < 0.05, ** *p* < 0.01, *** *p* < 0.001).

Next, the differential expression of the transporters between hepatocytes and NPCs as well as between the two NPC fractions was investigated. Concerning the first comparison, 13 genes were found to be differentially expressed (Figure 3). Here, Abcc1/Mrp1 (87×), Abcc4/Mrp4 (4×), Abcc5/Mrp5 (40×), Slc15a2/PepT2 (16×), Slc28a2/Cnt2 (20×), Slco3a1/Oatp3a1 (15×), and Slco4a1/Oatp4a1 (13×) showed significantly higher expression levels in NPCs, while Abcc2/Mrp2 (6×), Abcg2/Bcrp (6×), Slc19a2/ThTr1 (8×), and Slc22a1/Oct1 (7×) were expressed significantly higher in hepatocytes. No significant differences in the expression between Kupffer cells and endothelial cells were observed for the seven genes with higher expression in NPCs than in hepatocytes. However, mRNA levels of Abcb1a/Mdr1a (5×), Abcc3/Mrp3 (6×), Slc19a2/ThTr1 (2×), and Slc22a1/Oct1 (5×) were significantly higher in Kupffer cells (Figure 4).

Regarding nuclear receptors, the expression levels of Nr1i2/Pxr and Nr1i3/Car were significantly elevated in hepatocytes, showing a 20-fold and 5-fold increase, respectively, compared to the non-parenchymal cells (NPCs). In contrast, Kupffer cells exhibited a 3-fold higher expression level than endothelial cells.

In a further analysis, the expression in hepatocytes, Kupffer cells, and endothelial cells was directly compared (Supplemental Figures S3–S7). The analysis reveals that Abcc3/Mrp3 is 4-fold higher expressed in Kupffer cells, while Abcb1b/Mdr1b is 5-fold and 11-fold higher expressed in Kupffer cells and endothelial cells. In addition, Slc29a2/Ent2 (3-fold) and Slco4a1/Oatp4a1 (6-fold) are also more highly expressed in Kupffer cells. The other significantly differently expressed genes between Kupffer cells and endothelial cells (Abcb1a/Mdr1a, Slc19a2/ThTr1, Slc22a1/Oct1, and Nr1i3/Car) are expressed at much lower levels in both compared to hepatocyte expression.

## 3. Discussion

The aim of the study was to compare the expression of selected pharmacologically relevant drug transporters in non-parenchymal liver cells (NPCs) and parenchymal cells (PCs). For this purpose, these two cell types were first separated from each other using density gradient centrifugation. The endothelial cells (ECs) and Kupffer cell fractions (KCs) were then isolated from the NPCs using an antibody-based magnetic sorting method.

Further analyses were carried out at the RNA level using RT-qPCR, whereby the purity of the respective cell fractions was examined first. Abcb11/Bsep and Slc10a1/Ntcp were used as marker genes for hepatocytes [26,27,28,29] as well as Tie1 [30,31], Postn [32,33], and Clec4f [34,35,36] to identify the non-parenchymal endothelial cells, stellate cells, and Kupffer cells, respectively. The results showed a significantly stronger expression of the endothelial cell, Kupffer cell, and stellate cell markers in the NPC fraction compared to hepatocyte, while the opposite was true for the hepatocyte markers. This indicates a very efficient separation of parenchymal cells and non-parenchymal cells using centrifugation, which was comparable to the literature data [37,38]. The antibody-based isolation of endothelial cells and Kupffer cells from the NPC fraction was also comparable with other methods [39,40], although the degree of enrichment here was significantly lower compared to hepatocytes and NPCs in general. Overall, it can be concluded that the quality and purity of the respective cell fractions appear to be suitable for the subsequent analyses. However, when interpreting the corresponding results, it must be considered that, based on the mRNA expression analyses, the Kupffer cells and endothelial cells are contaminated with around 10 and 5% of hepatocytes. The same applies to the NPC fraction, which is contaminated with 7% hepatocytes, and to the Kupffer cell and endothelial cell fractions, which each contain 18 and 8% of the other cell population, respectively.

Following the successful validation of the method, the focus of the work was on characterizing the expression of various drug transporters and nuclear receptors in the respective liver cell fractions. Therefore, the mRNA expression of eight Abc-, twenty-one Slc-, and nine Slco-transporters, as well as the two most important nuclear receptors Pxr and Car, were analyzed. While the expression of genes like Abcb1b/Mdr1b, Slc15a2/PepT2, Slc22a3/Oct3, or Slco4a1/Oatp4a1 was very low or even absent in all analyzed fractions, the expression of other genes showed significant differences. In addition to Abcb11/Bsep and Slc10a1/Ntcp which are already used as hepatocyte markers, a predominant expression in the hepatocytes was also shown for Slc22a1/Oct1, Abcc2/Mrp2, Abcg2/Bcrp, Slc19a2/ThTr1, and the two nuclear receptors Nr1i2/Pxr and Nr1i3/Car, which was to be expected based on the literature data [18,41,42,43] and thus additionally emphasizes the validity of the isolation method. It is interesting in this context that such a difference could not be shown to the same extent for Slco1b2/Oatp1b2, which is the homologue to human SLCO1B1 and SLCO1B3, although this would have been expected [44,45]. In comparison with the expression data of Abcb11/Bsep and Slc10a1/Ntcp, this observation cannot be explained by contamination of the corresponding cell fractions with hepatocytes, so at least a low level of Slco1b2/Oatp1b2 expression must also be assumed in non-parenchymal liver cells.

On the other hand, transporters such as Abcb1b/Mdr1b, Abcc1/Mrp1, Abcc5/Mrp5, Slc15a2/PepT2, Slc28a2/Cnt2, Slco3a1/Oatp3a1 are almost exclusively or predominantly (Abcc4/Mrp4 and Slco4a1/Oatpa41) expressed in the non-parenchymal liver cell fraction or in the endothelial cell and Kupffer cell fraction (Figure 3 and Supplemental Figures S4–S6). Significant differences between the endothelial cells and Kupffer cells were also observed for Abcb1a/Mdr1a, Abcc3/Mrp3, Slc22a1/Oct1, Slc19a2/ThTr1, and Nr1i3/Car, whereby the expression in the Kupffer cells was increased in all cases. However, since the latter three genes in particular are expressed much more strongly in hepatocytes than in the NPCs and the Kupffer cell fraction is more heavily contaminated with hepatocytes than the endothelial cell fraction, it must be assumed that the observation for Slc22a1/Oct1, Slc19a2/ThTr1, and Nr1i3/Car cannot be attributed to the Kupffer cells themselves.

While the majority of studies to date have focused on the expression of drug transporters in hepatocytes as the relevant functional unit of the liver, corresponding studies on non-parenchymal cells are limited. However, our observation on the expression of Abcc1/Mrp1, Abcc3/Mrp3, Slc15a2/PepT2, Slc28a2/Cnt2, Slco3a1/Oatp3a1, and Slco4a1/Oatp4a1 in Kupffer cells is in line with the available literature data [43] and with results on transporter expression in macrophages [46,47,48,49]. The same applies to the expression of Abcc4/Mrp4, Abcc5/Mrp5, Slco3a1/Oatp3a1, and Slco4a1/Oatp4a1in liver endothelial cells [43], or endothelial cells in general [47,50,51].

Concerning Abcb1/Mdr1, it is established that in contrast to the human situation, there are two isoforms present in rodents, namely 1a and 1b [52,53,54]. Both were detected in various tissues including the liver [55]. The present study indicates that in the liver, the Abcb1a/Mdr1a isoform is predominantly expressed in hepatocytes, while Abcb1b/Mdr1b is dominant in NPCs (Supplemental Figure S4). However, the overall expression of Abcb1b/Mdr1b is relatively low compared to Abcb1a/Mdr1a. This finding was in contrast to a previous study demonstrating similar hepatic expression levels of both isoforms [56].

In the study, we concentrated on the two main fractions of non-parenchymal liver cells, Kupffer cells, and endothelial cells, although the transporter expression in the remaining NPC fractions, in particular the stellate cells and cholangiocytes, would certainly also be of interest. For example, Abcc4/Mrp4 has already been shown to be expressed not only in endothelial cells but also in stellate ones [43]. These findings indicate that NPCs should be incorporated into preclinical studies as well. A series of experiments have already been conducted on methotrexate and its effect on hepatocytes and NPCs, indicating a higher uptake into NPCs and an interplay between the different cell types [57,58]. Furthermore, co-culture studies of hepatocytes and different NPC fractions indicate increased sensitivity and altered metabolic activity of co-cultured cells with regard to drugs like paracetamol, troglitazone, or diclofenac [59,60]. In addition to these literature data, our own preliminary functional studies also confirm our findings. For example, the OCTN2/SLC22A5 substrate carnitine is taken up in both NPCs and hepatocytes, while 5-fluorouracil (5-FU), a substrate of the efflux transporter MRP5/ABCC5, is taken up significantly less efficiently in NPCs (high expression of ABCC5 in contrast to hepatocytes) (Supplemental Figure S8).

Our approach using a conventional qPCR method without pre-amplification is rather unfavourable for very rare NPC fractions like stellate cells with a proportion of around 1% due to the limited RNA yield, so that methods such as single cell analysis appear to be more suitable in this context [61]. Another limitation of our study is that we focused exclusively on the RNA level, meaning that the results must be verified in further studies at the protein and functional levels.

## 4. Materials and Methods

### 4.1. Primary Cell Isolation

**Figure 5:**
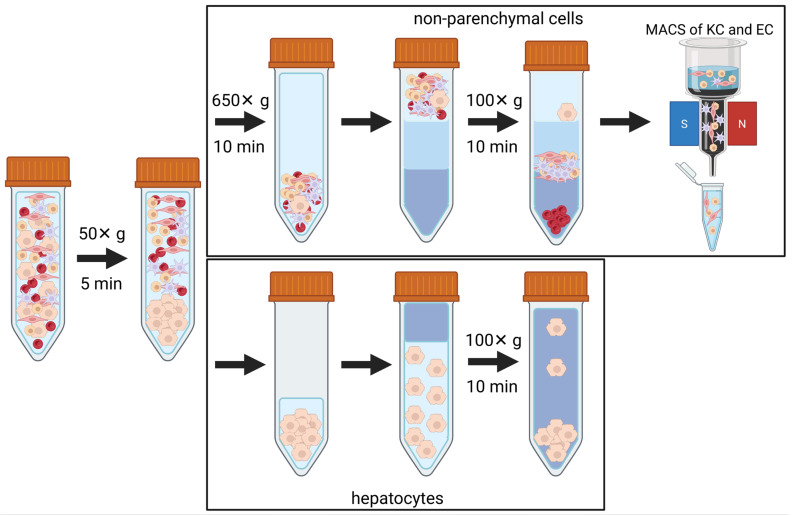
Schematic overview of the isolation protocol for hepatocytes and non-parenchymal cells (NPCs) after in vivo collagenase perfusion. The living hepatocytes and NPCs were separated through various centrifugation steps. Kupffer cells and endothelial cells were isolated using magnetic-activated cell sorting (MACS) and specific antibodies against Kupffer cells (F4/80) and endothelial cells (CD146) (the graphic was created using BioRender by Rönnpagel, V. (2025) https://BioRender.com/d13w873, accessed on 11 November 2025).

Primary hepatocytes and NPCs were isolated from CD-1 mice livers according to a protocol published by Seglen et al. [62]. We used animals of both sexes aged 2 to 3 months and weighing 30 to 40 g. The original protocol was modified with a Ca^2+^-containing perfusion buffer to ensure optimal collagenase functionality. This modification has been shown to be effective for the isolation of hepatocytes and NPCs [63,64,65]. In brief, mice were treated with ketamine (100 µg/g; Selectavet Dr. Otto Fischer GmbH, Weyarn, Germany) and xylazine (25 µg/g; Selectavet Dr. Otto Fischer GmbH, Weyarn, Germany). Following this, the liver was prepared and perfused via the portal vein with HBSS (Hanks Balanced Salt Solution (Merck, Darmstadt, Germany); supplemented with 0.1% EGTA (Carl Roth, Karlsruhe, Germany) for the first 5 min of perfusion, perfusion rate 5 mL/min) using a Surflo^®^-i.v. Catheter (Greiner BIO-ONE, Kremsmünster, Austria). To extract the liver cells, a final perfusion was performed using a hepatocyte isolation solution (Primacyt Cell Culture Technology, Schwerin, Germany) supplemented with collagenase type IV (90 µg/mL, Sigma-Aldrich, Burlington, MA, USA) for an additional 4 to 6 min. After digestion, the liver was excised. Cells were carefully removed from the remaining tissue by filtration through a cell strainer (MACS SmartStrainer (100 µm), Miltenyi Biotec, Bergisch Gladbach, Germany). Hepatocytes were separated from the cell solution by centrifugation (50× *g*; 5 min, 4 °C). While the hepatocytes sedimented in this step, the NPCs remained in the supernatant. The NPCs were isolated from the supernatant by a further centrifugation step (650× *g*, 10 min, 4 °C) and purified from blood cells and the remaining hepatocytes by centrifugation through a 25% and 50% Percoll (Sigma-Aldrich, Burlington, MA, USA) gradient (1800× *g*, 15 min, 4 °C, without using a brake). The NPCs accumulated in the interphase and were subsequently washed with HBSS (650× *g*, 10 min, 4 °C; Merck, Darmstadt, Germany). To isolate Kupffer cells and endothelial cells, the isolated NPCs were incubated with a Kupffer cell-specific F4/80 antibody according to the manufacturer’s protocol (Miltenyi Biotec, Bergisch Gladbach, Germany) for 15 min. After washing, the cells were centrifuged at 650× *g* for 7 min. The pellet was resuspended (MACS BSA Stock Solution diluted 1:20 with autoMACS^®^ Rinsing Solution, Miltenyi Biotec, Bergisch Gladbach, Germany) before F4/80-positive cells were separated using the MiniMACS™ system in combination with the MS columns (Miltenyi Biotec, Bergisch Gladbach, Germany). The procedure was repeated with the eluate containing the remaining F4/80-negative cells using endothelial cell-specific CD146 antibodies (Figure 5). All handling and treatment of living animals were performed in accordance with the local and national guidelines and regulations.

### 4.2. Cell Viability

Viability and cell count of hepatocytes were determined by trypan blue (CHEMAPOL, Prague, Czech Republic) as a surrogate for the quality of the preparation. Isolated hepatocytes were treated with trypan blue in a 1:1 ratio (100 µL of each) and analyzed using a Neubauer cell chamber (1 × 1 mm squares) (VWR International, Radnor, PA, USA). If the viability was below 80%, dead cells were removed by gradient (25% Percoll) centrifugation. Preparations with a viability below 40% were not subjected to further processing. The cell count of NPCs was determined in the same way.

### 4.3. RNA Expression

RNA of the respective cell fractions was isolated using TRIzol™ Reagent (Invitrogen, Waltham, MA, USA) according to the manufacturer’s protocol. RNA concentration was quantified using a NanoDrop 1000 (Thermo Fisher Scientific, Waltham, MA, USA) and cDNA synthesis was performed by the High Capacity RNA-to-cDNA assay (Life Technologies, Carlsbad, CA, USA) using 125 to 500 ng RNA. Gene expression analysis was performed using TaqMan^®^ low density array cards (TLDA, 48 gene format) and the TaqMan^®^ Gene Expression Master Mix (Life Technologies, Carlsbad, CA, USA) according to the manufacturer’s protocol (for the gene list see Supplemental Figure S1). For each of the 8 sample slots, 125 ng reverse-transcribed RNA was used. The experiment was conducted on a 7900 HT system (Applied Biosystems/Life Technologies, Carlsbad, CA, USA) with the standard temperature profile (50 °C for 2 min, 95 °C for 10 min, followed by 40 cycles of 97 °C for 30 s and 60 °C for 1 min).

### 4.4. Data Analysis

A total of 16 independent liver cell preparations were carried out for the study. Due to cell viability and purity of the respective cell preparations, hepatocyte, NPC, Kupffer, and endothelial cell fractions were not available from each liver. Therefore, the sample numbers are varied and no paired analyses were performed.

TLDA data were mainly used for the analysis of transporter expression. The analysis was performed using a fixed threshold value for all genes to allow a comparison of the respective expression levels. For Slc15a1/PepT1, Slc22a6/Oat1, Slc22a8/Oat3, Slc22a16/Oct6, Slc47a2/Mate2, and Slc51a/Ostα, no Ct-values (Ct = 40) were detected in at least 60% of the samples of the respective cell fractions and therefore excluded from further analyses. For Slc19a3/ThTr2, Slc22a2/Oct2, Slco1c1/Oatp1c1, and Slco5a1/Oatp5a1, only one fraction was excluded from further analyses for this reason. In all other cases, samples with Ct-value of 40 were included in further analysis. The data analysis was carried out using the delta-delta Ct-method.

For normalization, we measured the expression of glyceraldehyde 3-phosphate dehydrogenase (Gapdh), TATA-binding protein (Tbp), and 18SrRNA and checked the variance of the respective results (Supplemental Figure S2). For further normalization, the geometric mean of Gapdh and Tbp was used, because the variance of the 18S rRNA levels was much higher (variance: 23% for 18S rRNA vs. 8% and 6% for Gapdh and Tbp, respectively).

In a second step, the delta Ct-values were normalized to the respective value of whole liver (hepatocytes and NPCs) or NPCs (endothelial and Kupffer cells). For quality control purposes, relevant markers such as C-type lectin domain family 4 member (Clec4) for Kupffer cells [35], tyrosine kinase with immunoglobulin and EGF homology domains 1 (Tie1) for endothelial cells [66], Periostin (Postn) for stellate cells [32], and the transporters solute carrier family 10 member 1 (SLC10A1), as well as ATP-binding cassette subfamily B member 11 (ABCB11) for hepatocytes [67,68] were used to characterize the respective cell fractions. Data were presented as box plots, with the whiskers indicating the 10th to 90th percentiles. For statistical analysis, first the ROUT outlier test (Q-value of 2%) was performed, before differences between groups were analyzed using the Mann–Whitney test. Statistical significance was defined as *p* < 0.05 (*), *p* < 0.01 (**), and *p* < 0.001 (***). In addition, the differences between hepatocytes and Kupffer and endothelial cells were analyzed using the Kruskal–Wallis test with Dunn’s post hoc test (testing against hepatocytes). All statistical tests, as well as preparation of graphs, were performed using GraphPad Prism Version 8.0.2 (GraphPad Software, Boston, MA, USA).

## 5. Conclusions

In summary, our study shows that RT-qPCR is generally suitable for characterizing transporter expression in liver cell fractions. It also shows that several drug transporters are preferentially expressed in non-parenchymal cells, such as Kupffer cells and endothelial cells in particular. The extent to which this expression has an influence on hepatotoxic effects of certain compounds must be shown in further studies.

## Data Availability

The original contributions presented in this study are included in the article/Supplementary Materials. Further inquiries can be directed to the corresponding author.

## Supplementary Materials

The following supporting information can be downloaded at: https://www.mdpi.com/article/10.3390/ijms262211116/s1.
