# Supplementary material for: Expression of Selected Pharmacologically Relevant Transporters in Murine Non-Parenchymal Liver Cells Compared to Hepatocytes
Source: Int J Mol Sci. 2025 Nov 17;26(22):11116. doi: 10.3390/ijms262211116 (PMC12652808; doi:10.3390/ijms262211116)
Supplement: Supplementary file 1 [file ijms-26-11116-s001.zip › ijms-3937365-supplementary.pdf]

Sample 1		Sample 2		Sample 3		Sample 4		Sample 5		Sample 6		Sample 7		Sample 8	
Tbp	Slco3a1	Tbp	Slco3a1	Tbp	Slco3a1	Tbp	Slco3a1	Tbp	Slco3a1	Tbp	Slco3a1	Tbp	Slco3a1	Tbp	Slco3a1
Gapdh	Slco4a1	Gapdh	Slco4a1	Gapdh	Slco4a1	Gapdh	Slco4a1	Gapdh	Slco4a1	Gapdh	Slco4a1	Gapdh	Slco4a1	Gapdh	Slco4a1
Tie1	Slco5a1	Tie1	Slco5a1	Tie1	Slco5a1	Tie1	Slco5a1	Tie1	Slco5a1	Tie1	Slco5a1	Tie1	Slco5a1	Tie1	Slco5a1
Clec4f	Slc22a1	Clec4f	Slc22a1	Clec4f	Slc22a1	Clec4f	Slc22a1	Clec4f	Slc22a1	Clec4f	Slc22a1	Clec4f	Slc22a1	Clec4f	Slc22a1
Postn	Slc22a2	Postn	Slc22a2	Postn	Slc22a2	Postn	Slc22a2	Postn	Slc22a2	Postn	Slc22a2	Postn	Slc22a2	Postn	Slc22a2
Abcb11	Slc22a3	Abcb11	Slc22a3	Abcb11	Slc22a3	Abcb11	Slc22a3	Abcb11	Slc22a3	Abcb11	Slc22a3	Abcb11	Slc22a3	Abcb11	Slc22a3
Slc10a1	Slc22a4	Slc10a1	Slc22a4	Slc10a1	Slc22a4	Slc10a1	Slc22a4	Slc10a1	Slc22a4	Slc10a1	Slc22a4	Slc10a1	Slc22a4	Slc10a1	Slc22a4
Slco1b2	Slc22a5	Slco1b2	Slc22a5	Slco1b2	Slc22a5	Slco1b2	Slc22a5	Slco1b2	Slc22a5	Slco1b2	Slc22a5	Slco1b2	Slc22a5	Slco1b2	Slc22a5
Abcb1a	Slc22a6	Abcb1a	Slc22a6	Abcb1a	Slc22a6	Abcb1a	Slc22a6	Abcb1a	Slc22a6	Abcb1a	Slc22a6	Abcb1a	Slc22a6	Abcb1a	Slc22a6
Abcb1b	Slc22a7	Abcb1b	Slc22a7	Abcb1b	Slc22a7	Abcb1b	Slc22a7	Abcb1b	Slc22a7	Abcb1b	Slc22a7	Abcb1b	Slc22a7	Abcb1b	Slc22a7
18S rRNA	Slc22a8	18S rRNA	Slc22a8	18S rRNA	Slc22a8	18S rRNA	Slc22a8	18S rRNA	Slc22a8	18S rRNA	Slc22a8	18S rRNA	Slc22a8	18S rRNA	Slc22a8
Abcc1	Slc22a16	Abcc1	Slc22a16	Abcc1	Slc22a16	Abcc1	Slc22a16	Abcc1	Slc22a16	Abcc1	Slc22a16	Abcc1	Slc22a16	Abcc1	Slc22a16
Abcc2	Slc19a3	Abcc2	Slc19a3	Abcc2	Slc19a3	Abcc2	Slc19a3	Abcc2	Slc19a3	Abcc2	Slc19a3	Abcc2	Slc19a3	Abcc2	Slc19a3
Abcc3	Slc28a2	Abcc3	Slc28a2	Abcc3	Slc28a2	Abcc3	Slc28a2	Abcc3	Slc28a2	Abcc3	Slc28a2	Abcc3	Slc28a2	Abcc3	Slc28a2
Abcc4	Slc15a1	Abcc4	Slc15a1	Abcc4	Slc15a1	Abcc4	Slc15a1	Abcc4	Slc15a1	Abcc4	Slc15a1	Abcc4	Slc15a1	Abcc4	Slc15a1
Abcc5	Slc15a2	Abcc5	Slc15a2	Abcc5	Slc15a2	Abcc5	Slc15a2	Abcc5	Slc15a2	Abcc5	Slc15a2	Abcc5	Slc15a2	Abcc5	Slc15a2
Abcg2	Slc51a	Abcg2	Slc51a	Abcg2	Slc51a	Abcg2	Slc51a	Abcg2	Slc51a	Abcg2	Slc51a	Abcg2	Slc51a	Abcg2	Slc51a
Slco1a1	Slc51b	Slco1a1	Slc51b	Slco1a1	Slc51b	Slco1a1	Slc51b	Slco1a1	Slc51b	Slco1a1	Slc51b	Slco1a1	Slc51b	Slco1a1	Slc51b
Slco1a4	Slc47a1	Slco1a4	Slc47a1	Slco1a4	Slc47a1	Slco1a4	Slc47a1	Slco1a4	Slc47a1	Slco1a4	Slc47a1	Slco1a4	Slc47a1	Slco1a4	Slc47a1
Slc22a18	Slc47a2	Slc22a18	Slc47a2	Slc22a18	Slc47a2	Slc22a18	Slc47a2	Slc22a18	Slc47a2	Slc22a18	Slc47a2	Slc22a18	Slc47a2	Slc22a18	Slc47a2
Slc19a2	Nr1i2	Slc19a2	Nr1i2	Slc19a2	Nr1i2	Slc19a2	Nr1i2	Slc19a2	Nr1i2	Slc19a2	Nr1i2	Slc19a2	Nr1i2	Slc19a2	Nr1i2
Slco1c1	Nr1i3	Slco1c1	Nr1i3	Slco1c1	Nr1i3	Slco1c1	Nr1i3	Slco1c1	Nr1i3	Slco1c1	Nr1i3	Slco1c1	Nr1i3	Slco1c1	Nr1i3
Slco2a1	Slc29a1	Slco2a1	Slc29a1	Slco2a1	Slc29a1	Slco2a1	Slc29a1	Slco2a1	Slc29a1	Slco2a1	Slc29a1	Slco2a1	Slc29a1	Slco2a1	Slc29a1
Slco2b1	Slc29a2	Slco2b1	Slc29a2	Slco2b1	Slc29a2	Slco2b1	Slc29a2	Slco2b1	Slc29a2	Slco2b1	Slc29a2	Slco2b1	Slc29a2	Slco2b1	Slc29a2

reference genes	marker genes	transporter	nucleare receptor

Supplemental Figure S1. TLDA card plate assignment. In the plate format, 8 samples could be simultaneously measured with 48 different genes. The reference genes (n=3) are shown in green, the marker genes (n=5) are shown in blue, the transporter (n=38) are shown in orange and the nuclear receptors (n=2) are shown in yellow.

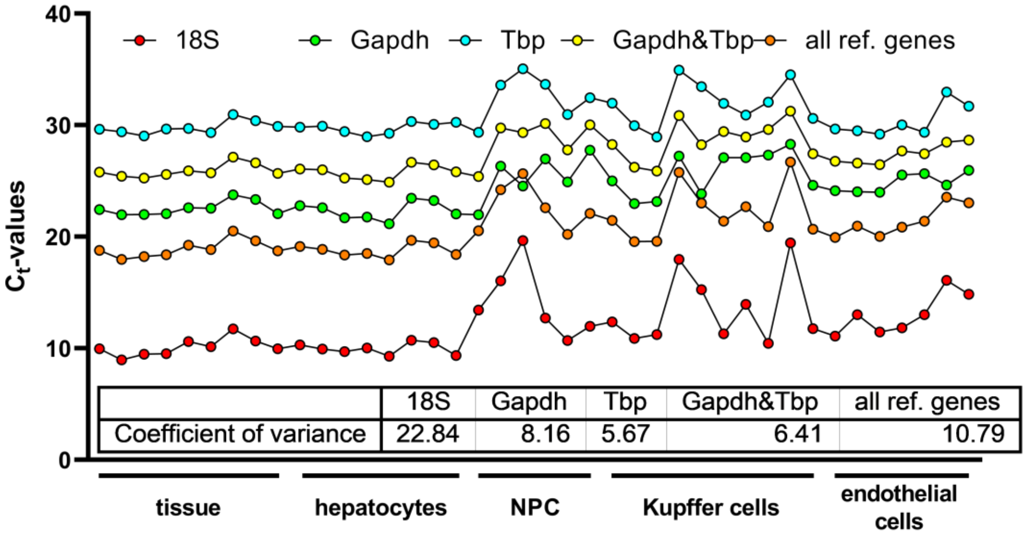

Supplemental Figure S2. Expression (Ct-values) of the measured reference genes in the respective samples (n=40). The coefficients of variation of the reference genes as well as reference gene pairs across all samples were also calculated.

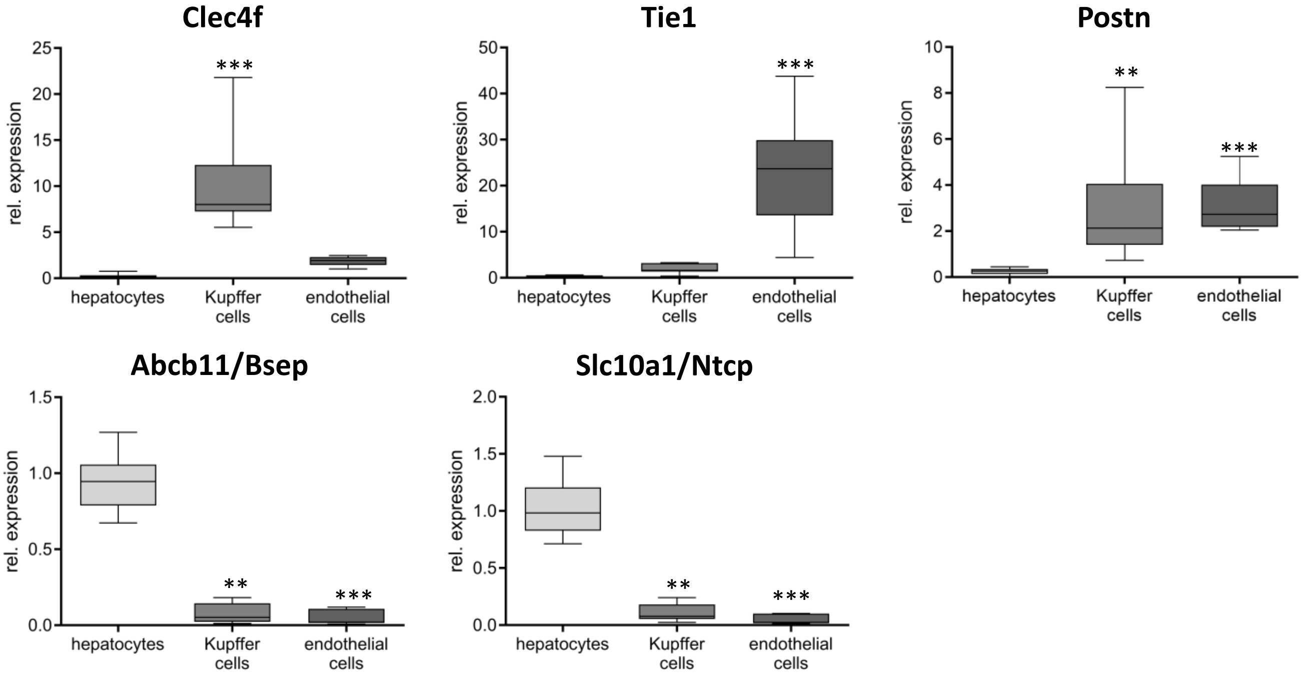

Supplemental Figure S3. Marker genes expression in hepatocytes, Kupffer cells and endothelial cells. The data were presented in relation to the expression in whole liver samples (n=9) after digestion. Statistical analyses were performed using the Kruskal-Wallis test in combination with Dunn´s multiple comparisons test (Kupffer cells (n=7-10) and endothelial cells (n=6-7) vs. hepatocytes (n=7-8); * $p < 0.05$, ** $p < 0.01$, *** $p < 0.001$).

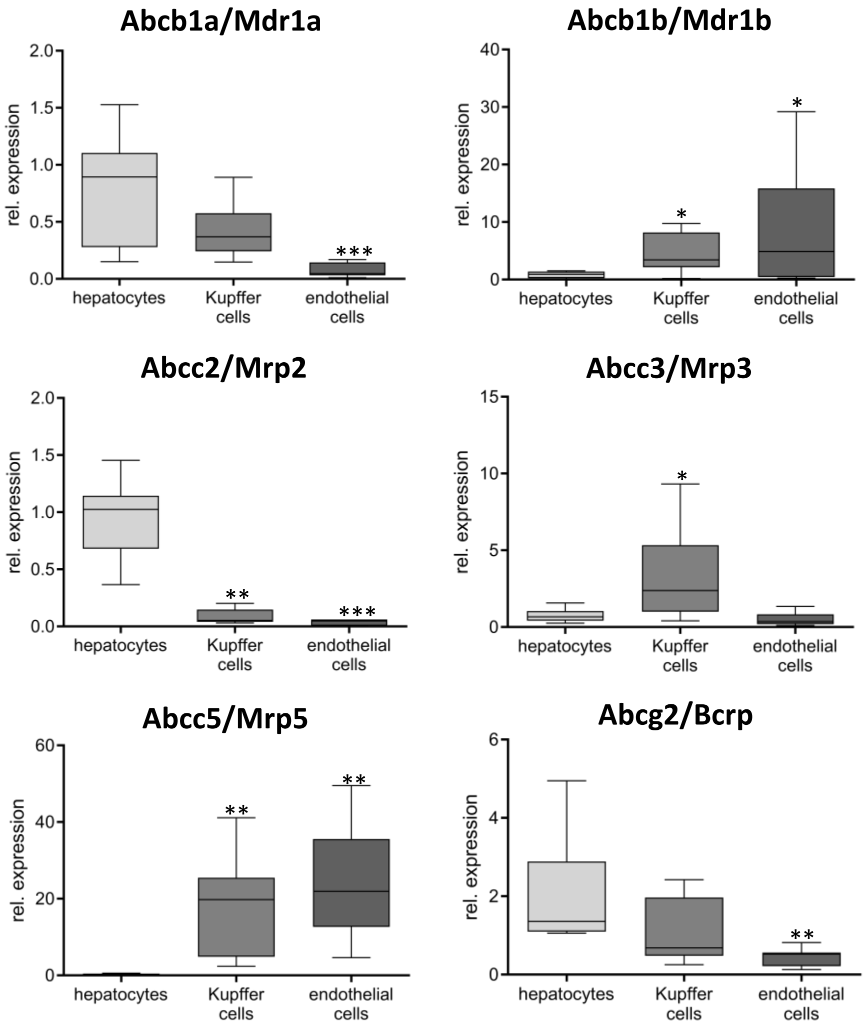

Supplemental Figure S4. Expression of Abc transporter in hepatocytes, Kupffer cells and endothelial cells. The data were presented in relation to the expression in whole liver samples (n=9) after digestion. Statistical analyses were performed using the Kruskal-Wallis test in combination with Dunn´s multiple comparisons test (Kupffer cells (n=7-10) and endothelial cells (n=7) vs. hepatocytes (n=7-8); * $p < 0.05$, ** $p < 0.01$, *** $p < 0.001$).

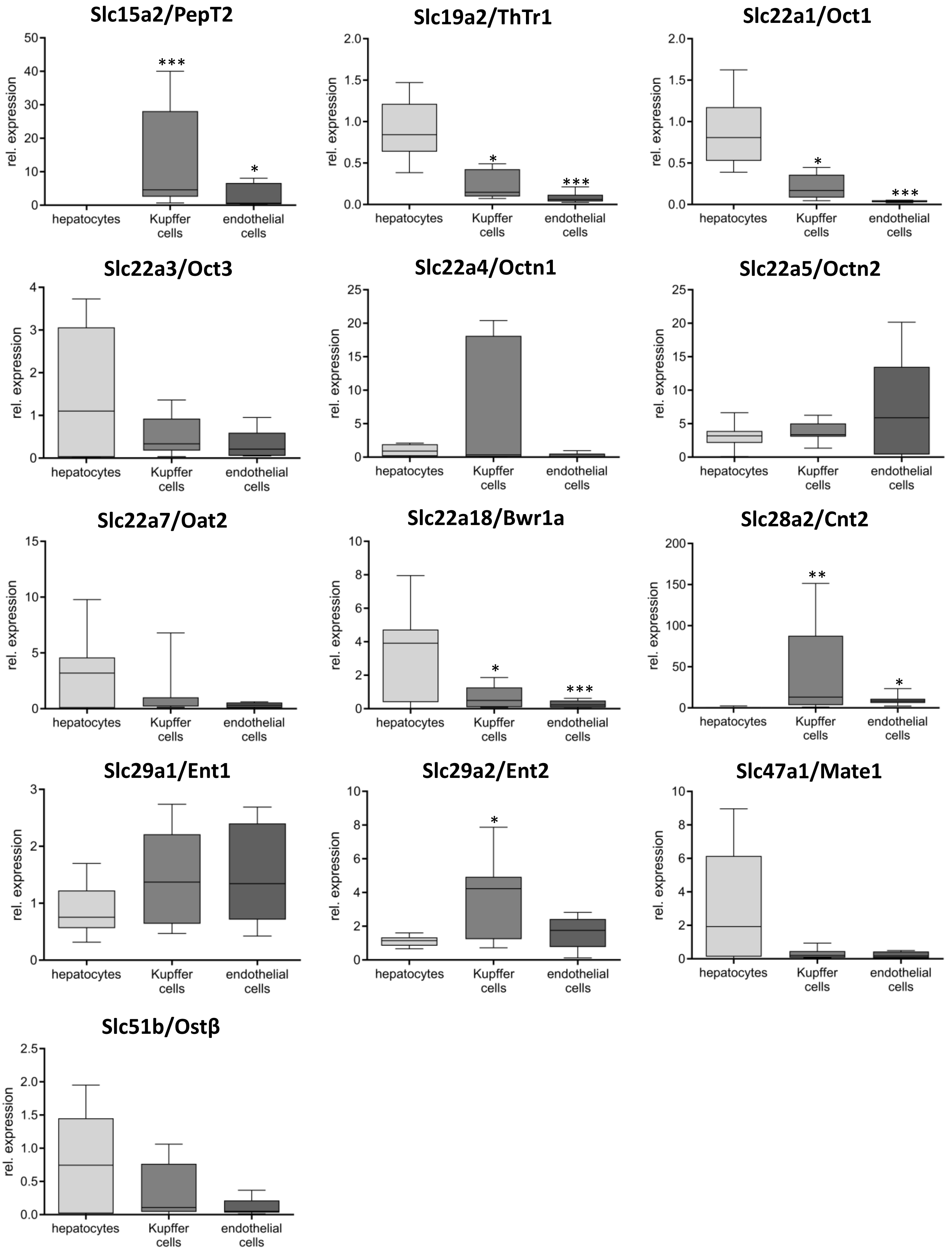

Supplemental Figure S5. Expression of Slc transporter in hepatocytes, Kupffer cells and endothelial cells. The data were presented in relation to the expression in whole liver samples (n=9) after digestion. Statistical analyses were performed using the Kruskal-Wallis test in combination with Dunn´s multiple comparisons test (Kupffer cells (n=6-10) and endothelial cells (n=5-7) vs. hepatocytes (n=6-8); * $p < 0.05$, ** $p < 0.01$, *** $p < 0.001$).

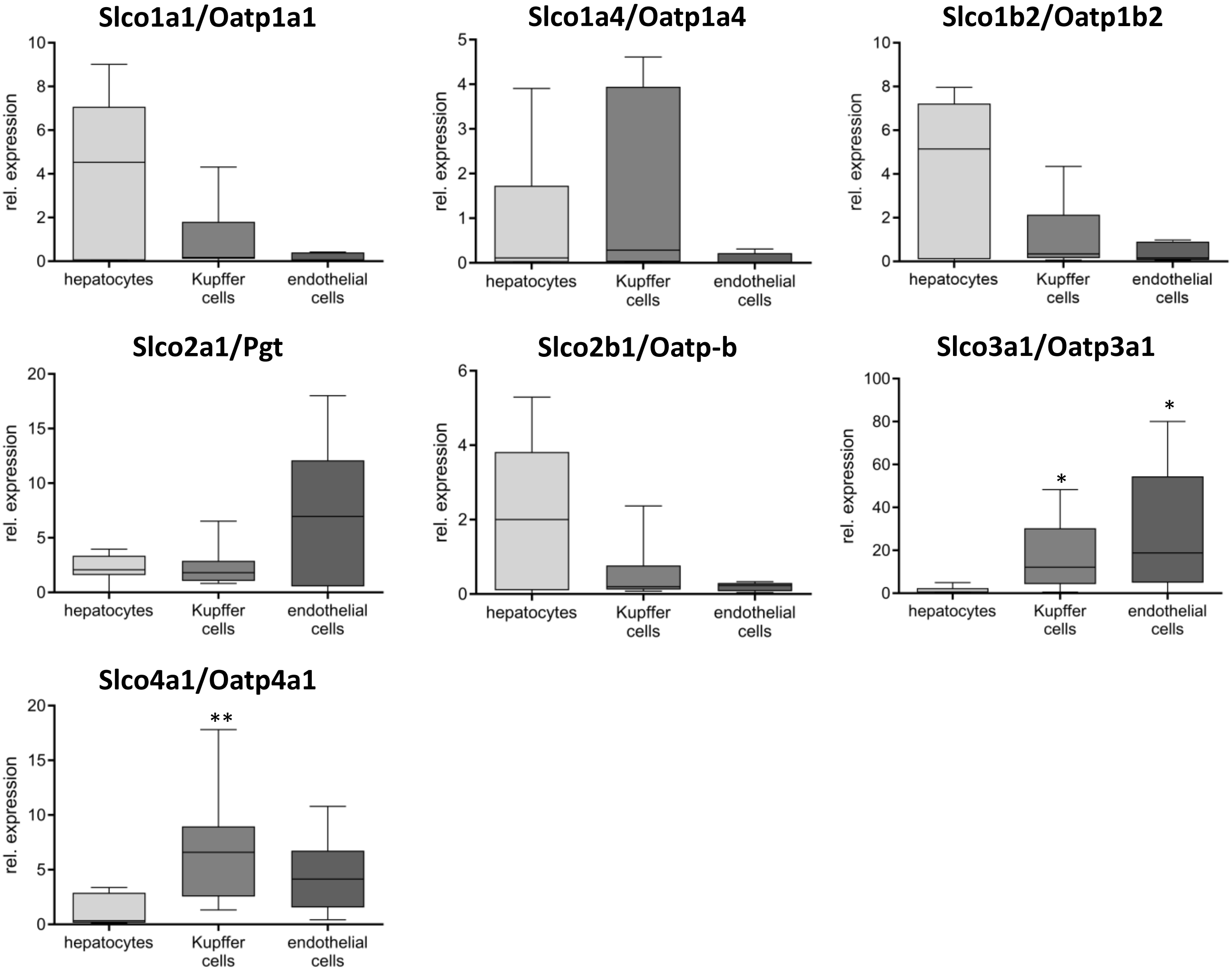

Supplemental Figure S6. Expression of Slco transporter in hepatocytes, Kupffer cells and endothelial cells. The data were presented in relation to the expression in whole liver samples (n=9) after digestion. Statistical analyses were performed using the Kruskal-Wallis test in combination with Dunn´s multiple comparisons test (Kupffer cells (n=7-10) and endothelial cells (n=5-7) vs. hepatocytes (n=6-8); * $p < 0.05$, ** $p < 0.01$, *** $p < 0.001$).

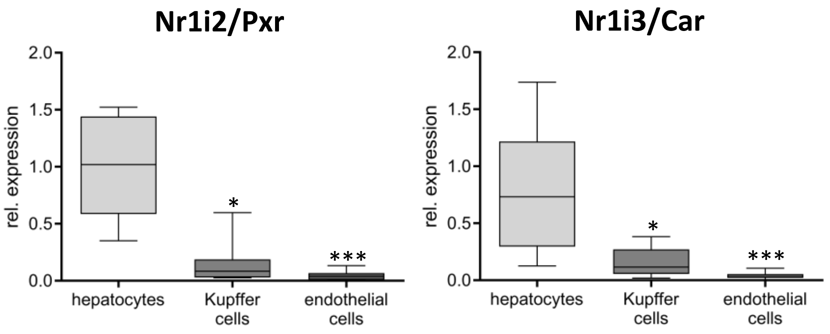

Supplemental Figure S7. Expression of nuclear receptors in hepatocytes, Kupffer cells and endothelial cells. The data were presented in relation to the expression in whole liver samples (n=9) after digestion. Statistical analyses were performed using the Kruskal-Wallis test in combination with Dunn´s multiple comparisons test (Kupffer cells (n=8-10) and endothelial cells (n=7) vs. hepatocytes (n=8); * $p < 0.05$, ** $p < 0.01$, *** $p < 0.001$).

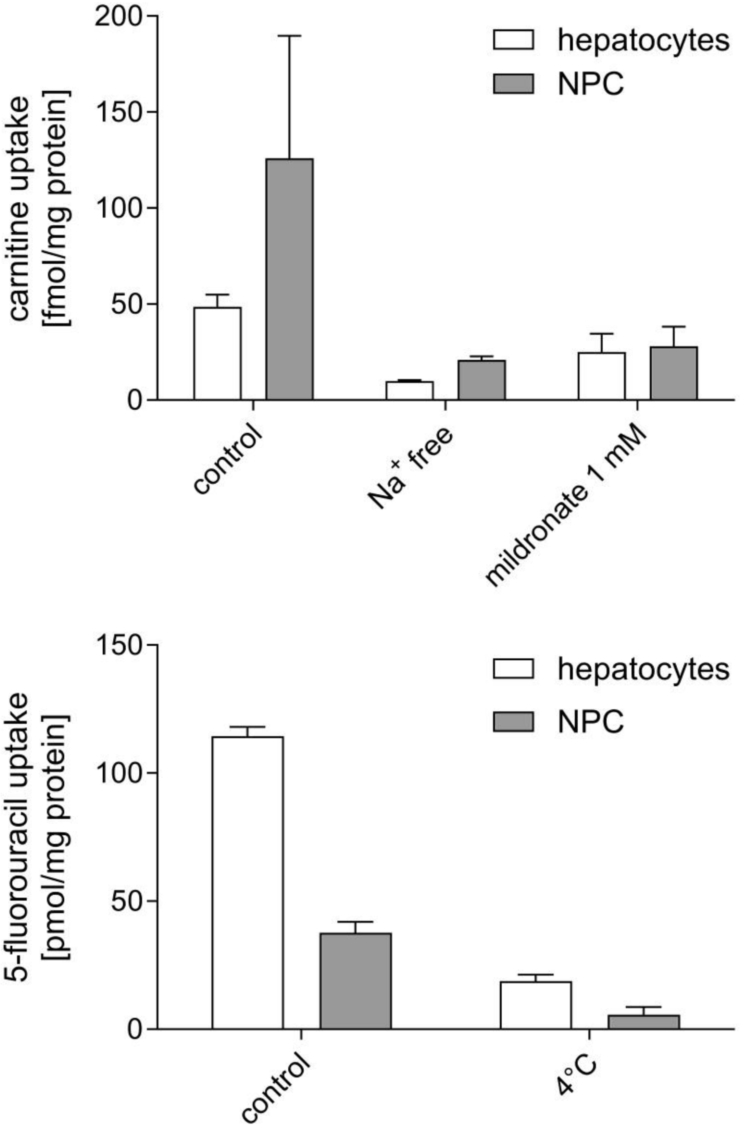

Supplemental Figure S8. Activity of the carnitine uptake transporter SLC22A5 (OCTN2) (upper diagramm) and the 5-fluorouracil efflux transporter (ABCC5(MRP5) (lower diagramm) was analyzed. Therefore, uptake assays (incubation conditions: 37°C for 30 min) were performed using suspension cells (hepatocytes and NPCs, 400,000 cells/reaction) and tritium transporter substrates (final concentrations: carnitine 12,5 nM, 1µCi/ml as well as 5-fluoruracil (18.7 µM,1µCi/ml), both ARC). To verify an OCTN2-dependent transport, sodium-free (replaced by choline) buffer as well as the OCTN2 inhibitor mildronate were used. In addition, the uptake of 5-fluoruracil was measured at 37°C and at 4°C for control. Uptake was normalized to protein concentration.
